# Proteomic stable isotope probing with an upgraded Sipros algorithm for improved identification and quantification of isotopically labeled proteins

**DOI:** 10.1186/s40168-024-01866-1

**Published:** 2024-08-08

**Authors:** Yi Xiong, Ryan S. Mueller, Shichao Feng, Xuan Guo, Chongle Pan

**Affiliations:** 1 School of Biological Sciences, University of Oklahoma, Norman, OK USA; 2 Department of Microbiology, Oregon State University, Corvallis, OR USA; 3 Department of Computer Science and Engineering, University of North Texas, Denton, TX USA; 4 School of Computer Science, University of Oklahoma, Norman, OK USA

## Abstract

**Background:**

Proteomic stable isotope probing (SIP) is used in microbial ecology to trace a non-radioactive isotope from a labeled substrate into de novo synthesized proteins in specific populations that are actively assimilating and metabolizing the substrate in a complex microbial community. The Sipros algorithm is used in proteomic SIP to identify variably labeled proteins and quantify their isotopic enrichment levels (atom%) by performing enrichment-resolved database searching.

**Results:**

In this study, Sipros was upgraded to improve the labeled protein identification, isotopic enrichment quantification, and database searching speed. The new Sipros 4 was compared with the existing Sipros 3, Calisp, and MetaProSIP in terms of the number of identifications and the accuracy and precision of atom% quantification on both the peptide and protein levels using standard *E. coli* cultures with 1.07 atom%, 2 atom%, 5 atom%, 25 atom%, 50 atom%, and 99 atom% ^13^C enrichment. Sipros 4 outperformed Calisp and MetaProSIP across all samples, especially in samples with ≥ 5 atom% ^13^C labeling. The computational speed on Sipros 4 was > 20 times higher than Sipros 3 and was on par with the overall speed of Calisp- and MetaProSIP-based pipelines. Sipros 4 also demonstrated higher sensitivity for the detection of labeled proteins in two ^13^C-SIP experiments on a real-world soil community. The labeled proteins were used to trace ^13^C from ^13^C-methanol and ^13^C-labeled plant exudates to the consuming soil microorganisms and their newly synthesized proteins.

**Conclusion:**

Overall, Sipros 4 improved the quality of the proteomic SIP results and reduced the computational cost of SIP database searching, which will make proteomic SIP more useful and accessible to the border community.

Video Abstract

**Supplementary Information:**

The online version contains supplementary material available at 10.1186/s40168-024-01866-1.

## Introduction

Stable isotope probing (SIP) is a molecular method to identify which microorganisms within a complex community are actively assimilating a specific substrate labeled with a stable isotope such as ^13^C, ^15^N, or ^2^H. It has been used to study the biomass decomposition processes in a variety of ecosystems, such as digestion of dietary nutrients by the mouse gut microbiome [1], degradation of lignocellulose by soil microorganisms [2], cycling of phytoplankton exudates by marine microbial communities [3]. SIP has also been used to identify the microbial guilds involved in the degradation of specific chemical compounds, including polybutylene succinate [4], aromatic hydrocarbon [5], polyvinyl chloride [6], and antibiotics [7]. Furthermore, SIP has been used to investigate the general metabolism of microbial communities, such as the slow-growing microbiomes in marine methane seep habitats [8] and the grassland microbial communities in warming and drought conditions [9].

A variety of SIP methods have been developed to trace a stable isotope from a labeled substrate into the biomass of microorganisms that have assimilated the substrate. Nucleic acid SIP (DNA- and RNA-SIP) involves the isolation of isotopically enriched DNAs or RNAs from SIP-labeled microorganisms using density-gradient ultracentrifugation [10, 11]. Sequencing of these nucleic acids can reveal the precise taxonomic structure and functional potential of the SIP-labeled microorganisms. Nucleic acid SIP is a commonly used SIP method owing to the wide availability of ultracentrifugation and sequencing. However, ultracentrifugation requires significant isotopic enrichment to separate out labeled nucleic acids, which necessitates high amounts of substrate addition and relatively long incubation times. The resulting fractions do not provide an accurate measurement of the enrichment levels of the extracted nucleic acids [12].

The isotopic labeling of phospholipid-derived fatty acids (PLFA) in microbial communities can be measured using gas chromatography-mass spectrometry (GC–MS) in a PLFA-SIP approach [13]. Unlike DNA- or RNA-SIP, the enrichment levels of PLFAs may be accurately quantified by mass spectrometry. However, labeled PLFAs can only be linked to very broad taxonomical categories of organisms, such as Gram-negative/positive bacteria, actinomycetes, and fungi [14]. This limitation prevents PLFA-SIP from identifying the precise microbial lineages labeled by SIP.

Labeled proteins in a microbial community provide an alternative target for SIP analysis. The first protein-based SIP is based on two-dimensional gel electrophoresis of an SIP-labeled proteome and a parallel unlabeled proteome [15]. Labeled protein spots are identified via the corresponding unlabeled protein spots on the same positions. Then, the enrichment levels of the labeled proteins were quantified based on the isotopic envelopes of their identified peptides. To take advantage of the higher throughput of shotgun proteomics, we subsequently developed a proteomic SIP approach [16] that can identify thousands of labeled proteins analyzed by liquid chromatography-tandem mass spectrometry (LC–MS/MS). Proteomic SIP uses enrichment-resolved database searching provided by the Sipros algorithm to identify peptide-spectrum matches (PSMs) and quantify their enrichment levels. Sipros-based proteomic SIP has been used to trace ^15^N and ^2^H in the acid mine drainage communities [17], ^15^N in the marine sediment communities [8], and ^13^C in the marine communities [3, 18]. These studies demonstrated some technical advantages of proteomic SIP over other SIP methods, including sensitive detection of labeled proteins at low abundance with low isotopic incorporation levels and accurate quantification of their enrichment levels. The labeled proteins can not only identify their source organisms with high taxonomic resolution but also reveal the de novo protein synthesis activities in these organisms during the assimilation of a given substrate.

However, our recent ^13^C SIP study of the soil communities [19] highlighted the high computational cost of SIP searches by Sipros 3 and the difficulty of finding labeled peptides from extremely complex communities. In this study, we upgraded the Sipros algorithm to overcome these two challenges. Sipros 4 was > 20-fold faster than Sipros 3 and identified more labeled proteins from SIP samples. Furthermore, we compared Sipros 4 with two other algorithms that can also be used for proteomics SIP, Calisp [20] and MetaProSIP [21]. The performances of these algorithms were benchmarked using standard ^13^C-labeled *E. coli* cultures with known enrichment levels and real-world ^13^C SIP soil communities.

## Results

### Validation of the proteomic SIP performance using standard *E. coli* proteomes

Peptides collected from triplicate *E. coli* cultures grown under ^13^C-SIP labeling conditions at six pre-defined atom% levels (i.e., 1.07 atom%, 2 atom%, 5 atom%, 25 atom%, 50 atom%, and 99 atom% ^13^C) were analyzed with liquid chromatography-tandem mass spectrometry (LC–MS/MS), which produced an average of 133,698 MS2 scans for each pre-defined atom% level. Sipros was used to compare each observed MS/MS spectrum with the theoretical MS/MS spectra predicted for each candidate peptide at the enrichment levels from 0 atom% ^13^C to 100 atom% ^13^C at 1% increments. The candidate peptides were generated by in silico digestion of protein sequences from the annotated *E. coli* genome. The best peptide-spectrum match (PSM) for an observed MS/MS spectrum identified both the peptide and its enrichment level.

**Fig. 1:**
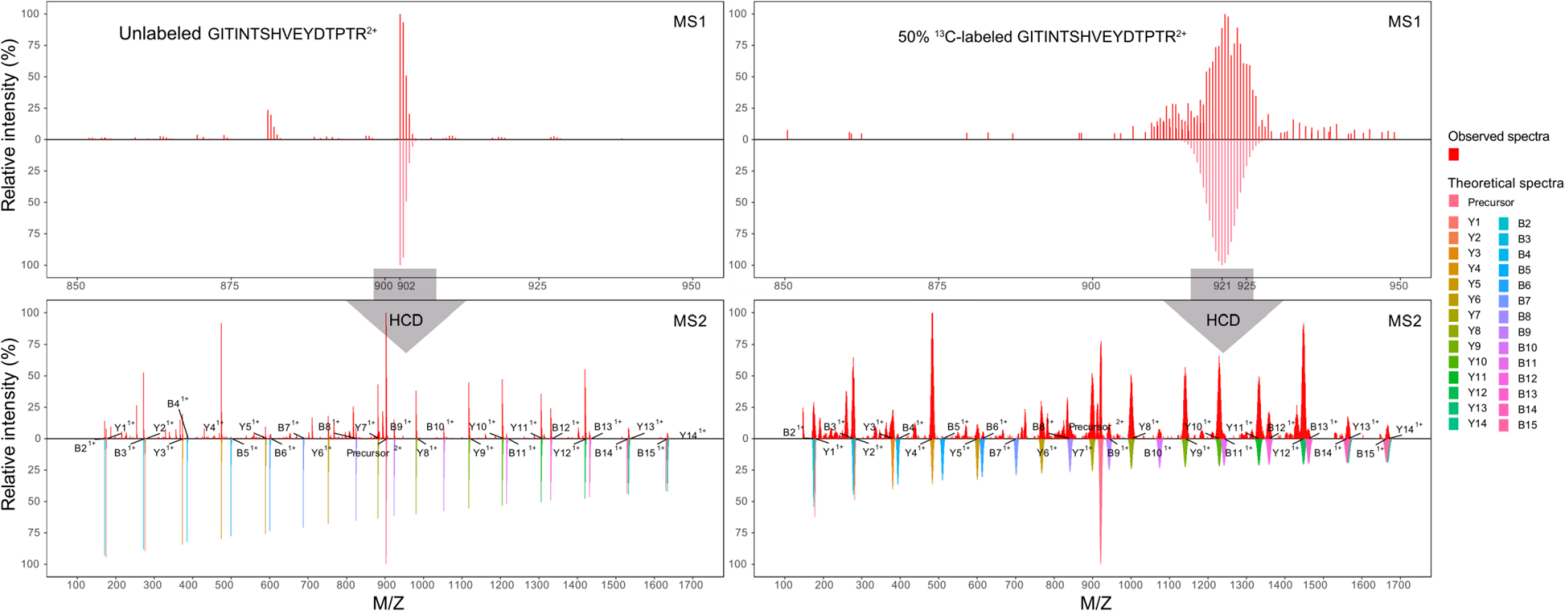
MS/MS measurement of a peptide’s unlabeled isotopologue with 1.07 atom% ^13^C and its labeled isotopologue with 50 atom% ^13^C. The MS1 scans (two upper panels) and the MS2 scans (two lower panels) are shown for the unlabeled isotopologue (two left panels) and the 50% ^13^C-labeled isotopologue (two right panels) of the peptide GITINTSHVEYDTPTR. Each panel shows both the observed spectrum (upper half) and the theoretical spectrum (lower half). The ^13^C labeling increased the m/z values and widened the isotopic envelopes of both the precursor ions in the MS1 scans and the product ions in the MS2 scans. The fragmentation pattern of the peptide was similar between its two isotopologues

Figure 1 shows the changes in the isotopic distributions of both the precursor and fragment ions of an illustrative peptide when its ^13^C level was enriched from the natural 1.07 atom% to 50 atom%. The higher ^13^C enrichment shifted and broadened the isotopic envelopes of not only the precursor ion in the MS1 scans but also the fragment ions in the MS2 scans. The 50-atom% ^13^C-labeled *E. coli* proteome was measured by LC–MS/MS at the five isolation window widths of 0.8, 1.5, 3.0, 5.0, and 7.0 Da. The 5-Da-wide isolation window produced the most peptide and protein identifications (Supplementary Table S1) and, therefore, was used to measure all the other standard *E. coli* proteomes.

**Fig. 2:**
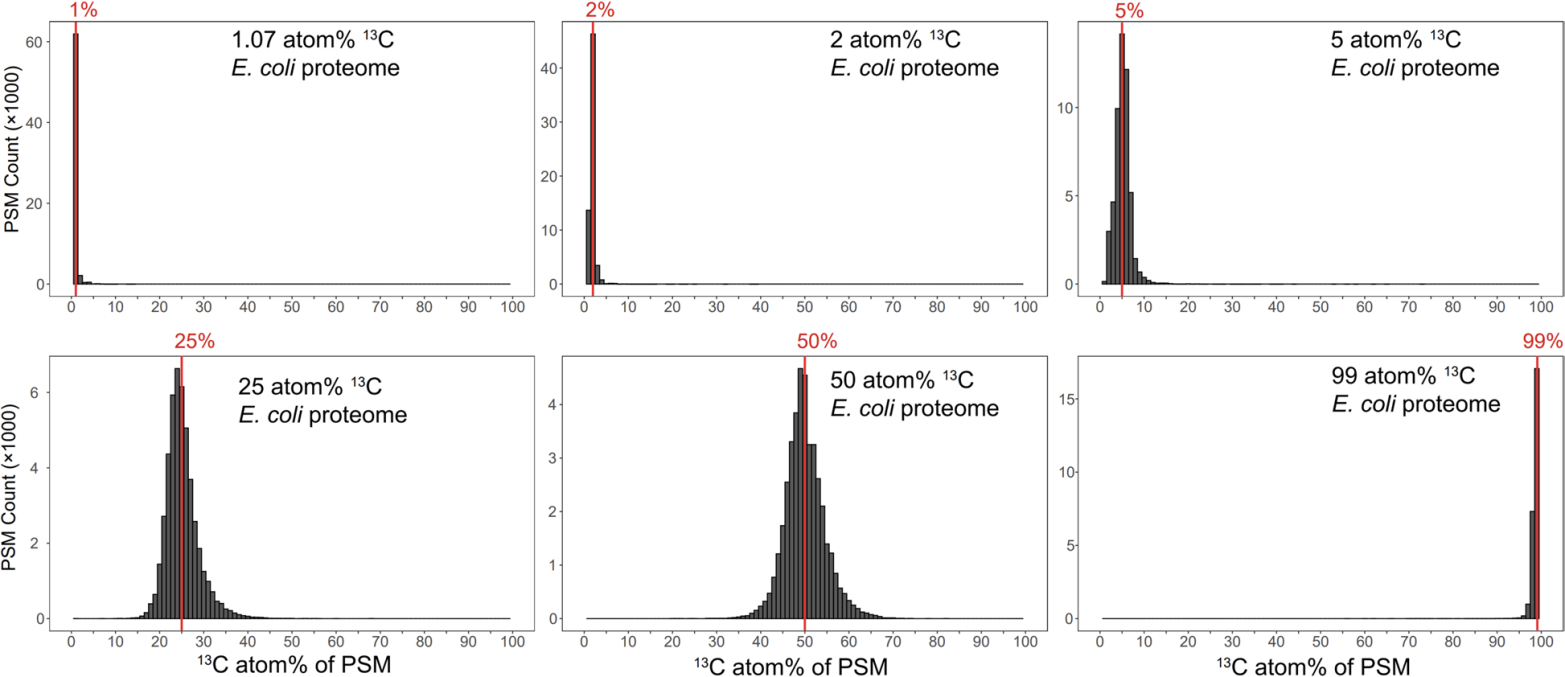
Accuracy and precision of ^13^C atom% quantification by Sipros 4 on the PSM level for standard *E. coli* samples. The atom% estimates for all PSMs identified by Sipros 4 in each *E. coli* proteome are shown in its corresponding histogram with 1-atom% bin width. The medians of the atom% histograms are exactly aligned to their expected atom% values marked by the red vertical line, which indicates accurate ^13^C atom% quantification by Sipros 4 across the full range of ^13^C enrichment levels. The dispersion of the atom% histograms measures the precision of ^13^C atom% quantification, which decreases gradually from the unlabeled sample to the 50% ^13^C-labeled sample and then increases in the 99% ^13^C-labeled sample. The size of atom% histograms reflects the number of identified PSMs, which ranges from 26,583 PSMs in the 99% ^13^C-labeled sample to 64,113 PSMs in the unlabeled sample

The ^13^C atom% estimates for PSMs identified by Sipros 4 from the standard *E. coli* proteomes showed strong concordance with their expected ^13^C enrichment levels (Fig. 2 and Supplementary Table S2). The median ^13^C atom% of the identified PSMs in all samples was equal to the expected ^13^C atom% from 1 to 99% ^13^C. This indicated accurate atom% quantification across the full range of ^13^C enrichment levels. The precision of atom% quantification decreased as the enrichment levels moved toward the 50 atom% ^13^C enrichment from the two ends (Fig. 2). Sipros 4 identified and enrichment-quantified 64,113 PSMs in the 1.07-atom% samples, 65,131 PSMs in the 2-atom% samples, 46,665 PSMs in the 5-atom% samples, 43,526 PSMs in the 25-atom% samples, 37,659 PSMs in the 50-atom% samples, and 26,583 PSMs in the 99-atom% samples. This indicated deep coverages of the labeled proteomes across the entire atom% range by Sipros 4.

### Comparison of the proteomic SIP performance using standard *E. coli* samples

Sipros 4 was compared with Sipros 3, Calisp, and MetaProSIP using the standard *E. coli* samples (Table 1). Because Calisp and MetaProSIP do not provide results at the PSM level, the performances of the four algorithms were compared at the peptide and protein levels. The comparisons used the three performance metrics described above, including atom% medians for quantification accuracy, atom% MADs for quantification precision, and identification counts for proteome coverage. Calisp, which needs peptides to be identified by Proteome Discoverer before quantifying their atom%, failed to function for the samples with 25 atom%, 50 atom% and 99 atom% ^13^C because Proteome Discoverer was unable to identify any protein in these samples (Supplementary Table S3) In comparison, Sipros 4 identified 973 proteins/protein groups with 25 atom% ^13^C, 893 proteins/protein groups with 50 atom% ^13^C, and 1493 proteins/protein groups with 99 atom% ^13^C. Moreover, Sipros 4 identified significantly more proteins/protein groups than Calisp in the 5%- and 2%-labeled samples and the unlabeled samples. Sipros 4 also identified higher numbers of proteins/protein groups than MetaProSIP in all samples (Table 1). The overlaps among the proteins identified by the three tools in these samples are shown in Supplementary Figure S1.

**Table 1:** Comparison of Sipros 3, Sipros 4, Calisp, and MetaproSIP on *E. coli* standard samples

Expected ^13^C atom%			1.07% ^13^C	2% ^13^C	5% ^13^C	25% ^13^C	50% ^13^C	99% ^13^C
Peptides^c^	Median of ^13^C atom%	Sipros 4	1.1%	2.0%	5.0%	25.0%	50.0%	99.0%
		Sipros 3	1.0%	1.0%	1.0%	25.0%	51.0%	99.0%
		Calisp	1.1%	2.0%	4.9%	NA^a^	NA	NA
		MetaProSIP	0.8%	1.2%	5.3%	10.2%	11.8%	97.7%
	MAD^b^ of ^13^C atom%	Sipros 4	0.0%	0.0%	1.5%	1.5%	3.0%	0.0%
		Sipros 3	0.0%	0.0%	0.0%	4.4%	5.2%	0.0%
		Calisp	1.2%	1.3%	1.5%	NA	NA	NA
		MetaProSIP	0.3%	0.1%	2.7%	5.7%	7.6%	1.0%
	Count	Sipros 4	18,147	14,523	11,297	8188	7275	9901
		Sipros 3	18,003	14,824	12,420	5478	2218	10,042
		Calisp	5259	6936	6137	NA	NA	NA
		MetaProSIP	11,695	10,131	5,741	1003	730	85
Proteins/protein groups^c^	Median of ^13^C atom%	Sipros 4	1.1%	2.0%	5.0%	25.0%	50.0%	99.0%
		Sipros 3	1.0%	1.0%	1.0%	25.0%	51.0%	99.0%
		Calisp	1.1%	2.0%	4.9%	NA	NA	NA
		MetaProSIP	0.8%	1.1%	2.1%	9.8%	11.2%	97.7%
	MAD^b^ of ^13^C atom%	Sipros 4	0.0%	0.0%	0.0%	1.5%	1.5%	0.0%
		Sipros 3	0.0%	0.0%	0.0%	1.5%	3.0%	0.0%
		Calisp	1.2%	1.3%	1.5%	NA	NA	NA
		MetaProSIP	0.1%	0.1%	1.5%	4.7%	6.2%	0.9%
	Count	Sipros 4	1815	1458	1210	973	893	1490
		Sipros 3	1834	1546	1428	871	476	1491
		Calisp	723	1105	973	NA	NA	NA
		MetaProSIP	1592	1429	1202	549	466	59

^a^*NA* data is not available because database searching failed to produce any identification

^b^*MAD* median absolute deviation

^c^FDRs at the peptide level and the protein level were controlled at 1%

Calisp produced accurate atom% medians in the three samples that it functioned with 1.07 atom%, 2 atom%, and 5 atom% ^13^C. MetaProSIP severely underestimated the atom% of proteins in the four samples other than the unlabeled and 99% ^13^C-labeled samples. The median atom% estimated by Sipros 4 were exactly aligned with the expected values in all six samples. Furthermore, the MAD of atom% estimates by Sipros 4 was lower than Calisp and MetaProSIP across all samples. This indicates that Sipros 4 excelled in quantifying the enrichment values across the full enrichment range.

Sipros 4 outperformed Sipros 3 in atom% quantification accuracy for the 2-atom% and 5-atom% ^13^C-labeled samples, as well as in the atom% precision and the proteome coverage for the 50-atom% sample. Notably, Sipros 4 identified approximately 2 and 4 times more PSMs than Sipros 3 at 25 atom% and 50 atom% ^13^C, respectively (Supplementary Table S2). The performance of Sipros 4 and Sipros 3 was also benchmarked using the previously analyzed ^15^N-labeled standards from the acid mine drainage biofilm community [22] (Supplementary Table S4). Sipros 4 identified 63% more PSMs than Sipros 3 in the 50-atom% ^15^N-labeled sample and the two algorithms performed similarly in the unlabeled sample and 98-atom% ^15^N-labeled sample.

In addition, Sipros 4 was tested using a ^15^N-labeled spiked mouse gut microbiome sample that was measured using low-resolution MS2 in an ion trap mass analyzer [23]. In the previous study, 5,945 ^15^N-labeled peptides were identified at 0.1 FDR using MetaProSIP [23]. Here, Sipros 4 identified 7,439 ^15^N-labeled peptides at 0.1 FDR and 3877 ^15^N-labeled peptides at 0.01 FDR (Supplementary Table S5). This dataset validated the performance of Sipros 4 using a spiked gut microbiome, although optimum identification results from Sipros 4 required high-resolution MS2 using a 5-Da isolation window.

The code optimization for Sipros 4 increased the computational efficiency of the enrichment-resolved database searching. We benchmarked the wall-clock time used by Sipros 4, Sipros 3, Calisp, and MetaProSIP for processing the standard *E. coli* datasets. All algorithms were run on the same computer server with 24 CPU cores. Sipros 4 used ~ 0.5 h and Sipros 3 used more than 12 h to search the MS/MS datasets at each atom% level (Supplementary Table S2). The search time of the same datasets with Calisp included ~ 2.5 h used by Proteome Discoverer for protein identification and ~ 0.5 h used by Calisp for ^13^C atom% quantification. The search time with MetaProSIP included ~ 0.5 h consumed by Comet for protein identification and a few minutes consumed by MetaProSIP itself for ^13^C atom% quantification. Thus, the upgrade of Sipros to version 4 reduced its computational cost to be on par with Calisp and MetaProSIP.

### Comparison of the proteomic SIP performance using ^13^C SIP soil communities

The performance of the four algorithms was benchmarked using a set of soil community samples analyzed in a previous SIP study where either ^13^C-methanol or ^13^CO_2_ was used as the labeling substrate [19]. These samples were chosen to test the performance of each algorithm on much more complex and diverse metaproteomes than the standard *E. coli* proteomes analyzed above. For each soil SIP experiment, the number of ^13^C-labeled identifications was summarized at the PSM level when available, the peptide level, and the protein level (Table 2). The initial soil samples collected prior to ^13^C-incubation should not contain any ^13^C-labeled protein and, thus, can be used as a negative control in which any ^13^C-labeled identifications should be considered as false positives. The four algorithms identified none or a single identification with ≥ 5 atom% ^13^C from the initial soil samples. This suggested a low false discovery rate among ^13^C-labeled identifications with ≥ 5 atom% by the four algorithms.

**Table 2:** Number of ^13^C-labeled PSMs, peptides, and proteins identified with ≥ 5 atom% ^13^C in the initial soil, ^13^C-methanol SIP soil, and ^13^CO2 SIP soil

SIP	Labeled organisms	Algorithms	# Labeled PSMs	# Labeled peptides^b^	# Labeled proteins/protein groups^b^
Initial soil (no labeling)	Microbes	Sipros 4	0	0	0
		Sipros 3	1	1	1
		Calisp	NA^a^	0	0
		MetaProSIP	NA^a^	0	0
	Plants	Sipros 4	0	0	0
		Sipros 3	0	0	0
		Calisp	NA	0	0
		MetaProSIP	NA	0	0
^13^C-methanol SIP	Microbes	Sipros 4	614	270	153
		Sipros 3	342	129	81
		Calisp	NA	18	15
		MetaProSIP	NA	13	13
^13^CO_2_ SIP	Microbes	Sipros 4	244	124	84
		Sipros 3	73	37	29
		Calisp	NA	19	19
		MetaProSIP	NA	1	1
	Plants	Sipros 4	161	36	26
		Sipros 3	69	20	15
		Calisp	NA	1	1
		MetaProSIP	NA	2	2

^a^*NA* data is not available because Calisp and MetaProSIP do not provide PSM identifications

^b^FDRs at the peptide level and the protein level were controlled at 1%

In the ^13^C-methanol SIP experiment, the initial soil samples were amended with ^13^C-labeled methanol. To identify proteins and microorganisms that incorporated ^13^C from methanol, enrichment-resolved database searching was performed against the microbial proteins identified in the unlabeled regular search (Supplementary Table S6). Sipros 4 identified 153 labeled proteins/protein groups with ≥ 5 atom% ^13^C based on 270 labeled peptides. In comparison, Sipros 3 identified 81 ^13^C-labeled proteins/protein groups based on 129 labeled peptides, Calisp identified 15 ^13^C-labeled proteins based on 18 labeled peptides, and MetaProSIP identified 13 ^13^C-labeled proteins based on 13 labeled peptides (Table 2). While most of the protein identifications by Calisp and MetaProSIP were based on a single peptide identification, Sipros 3 and Sipros 4 generated multiple peptide identifications for most protein identifications and provided two PSMs, on average, per peptide identification.

In the ^13^CO_2_ SIP experiment, the rhizosphere soil samples were collected from plants grown in the initial soil in a ^13^CO_2_-amended atmosphere. The extracted metaproteomes were searched against both the microbial proteins and the plant host proteins. From the rhizosphere microbial communities, Sipros 4 identified 244 ^13^C-labeled PSMs and 124 ^13^C-labeled peptides, which were assembled into 84 ^13^C-labeled proteins/protein groups (Table 2). In comparison, 29, 19, and 1 ^13^C-labeled microbial proteins/protein groups were identified by Sipros 3, Calisp, and MetaProSIP, respectively. Sipros 4 also identified 26 ^13^C-labeled plant proteins/protein groups, which was also much more than Sipros 3, Calisp, and MetaProSIP (Table 2).

### Biological analysis of the proteomic SIP results from ^13^C SIP soil communities

**Fig. 3:**
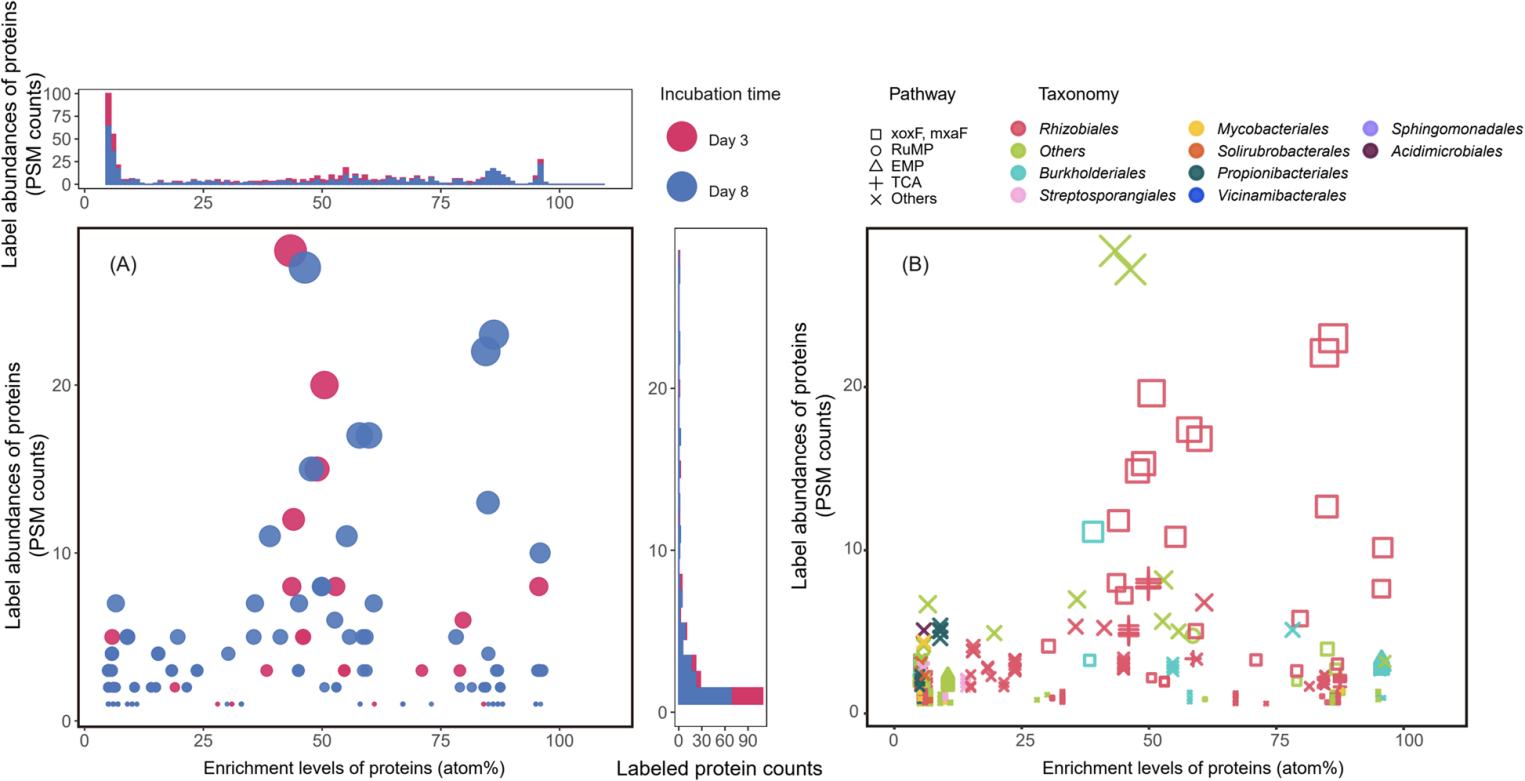
^13^C enrichment levels and label abundances of the proteins labeled by ^13^C-methanol SIP. Both proteomic SIP scatterplots show all the identified proteins with ≥ 5 atom% ^13^C by their enrichment levels (^13^C atom%) on the* x*-axis and their label abundances (labeled PSM counts) on the *y*-axis. The sizes of the data points are proportional to the labeled PSM counts of the proteins. **A** Comparison of the labeled proteins identified after 3 days of labeling (red solid circles) and 8 days of labeling (blue solid circles). The top histogram shows the distribution of the label abundances across the enrichment levels. The right histogram shows the distribution of labeled proteins by their label abundance. The day 8 sample contained more labeled proteins with higher label abundances at higher enrichment levels than the day 3 samples. **B** Taxonomy and functions of the labeled proteins. The colors of the symbols represent the taxonomy assignments at the order level of the labeled proteins. The shape of the symbols represents the metabolic pathway assignments of the labeled proteins. xoxF and mxaF are two methanol dehydrogenases. RuMP is the ribulose monophosphate pathway involved in the methanol assimilation. EMP is the Embden-Meyerhof-Parnas pathway for glycolysis. TCA is the tricarboxylic acid cycle. The functions of many of the labeled proteins are related to methanol metabolisms

For all the identified proteins in a proteomic SIP sample, Sipros 4 quantified both their isotopic enrichment levels in terms of atom% and their label abundances in terms of labeled spectral counts (Fig. 3). In the ^13^C-methanol SIP experiment, the soil community was sampled after 3 days and after 8 days of daily ^13^C-methanol addition. The ^13^C-labeled proteins identified in the day-3 and day-8 samples were shown by their enrichment levels and label abundances in Fig. 3. 186 ^13^C-labeled PSMs, 97 peptides, and 63 proteins/protein groups were identified in the day-3 samples. 428 ^13^C-labeled PSMs, 255 peptides, and 135 proteins/protein groups were identified in the day-8 samples. The median ^13^C enrichment levels of labeled proteins increased from 43.5 atom% on day 3 to 53.5 atom% on day 8 (Fig. 3). This indicated that, over the 5 additional days of ^13^C-methanol addition, a larger number of proteins were labeled, more copies of the labeled proteins were synthesized as indicated by their higher label abundances, and more ^13^C was incorporated into the labeled proteins as indicated by their higher enrichment levels.

**Fig. 4:**
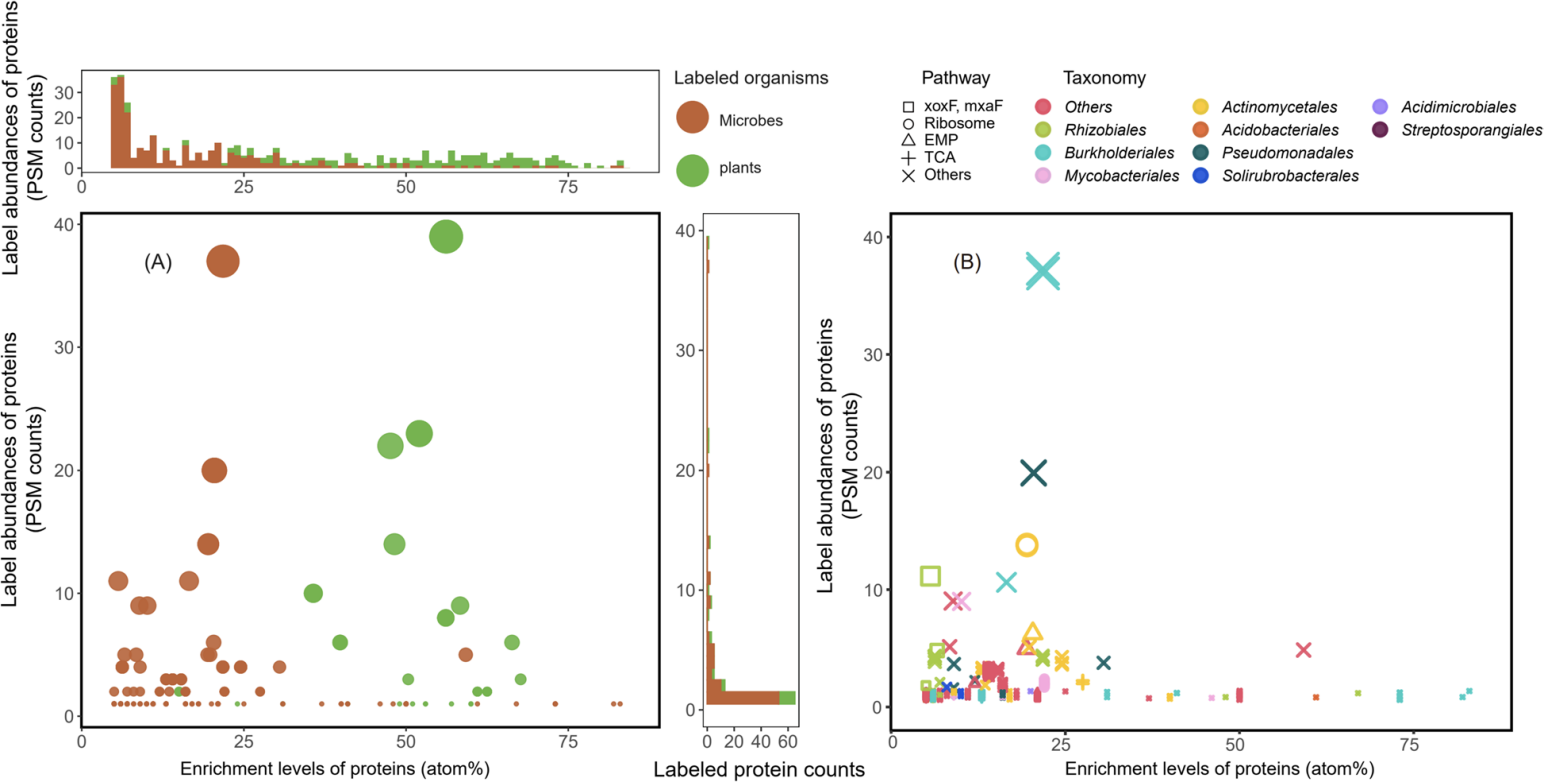
^13^C enrichment levels and label abundances of the proteins labeled by ^13^CO2 SIP. Both proteomic SIP scatterplots show all the identified proteins with ≥ 5 atom% ^13^C by their enrichment levels (^13^C atom%) on the *x*-axis and their label abundances (labeled PSM counts) on the *y*-axis. **A** Comparison of the labeled proteins identified from microorganisms (brown solid circles) and those from plants (green solid circles). The plant proteins were labeled at much higher enrichment levels than the microbial proteins. **B** Taxonomy and functions of the labeled microbial proteins. The colors of the symbols represent the taxonomy assignments at the order level of the labeled proteins. The shape of the symbols represents the metabolic pathway assignments of the labeled proteins. xoxF and mxaF are two methanol dehydrogenases. RuMP is the ribulose monophosphate pathway involved in the methanol assimilation. EMP is the Embden-Meyerhof-Parnas pathway for glycolysis. TCA is the tricarboxylic acid cycle

In the ^13^CO_2_ SIP experiment, ^13^C was expected to be fixed by the plants in their leaves, then transported to their roots, and finally transferred to the rhizosphere communities. Sipros 4 identified both rhizosphere microbial proteins and plant proteins from the rhizosphere soil samples (Fig. 4). The rhizosphere communities yielded 244 ^13^C-labeled PSMs, 124 labeled peptides, and 84 labeled proteins/protein groups. Because only a small amount of root materials may be present in the rhizosphere soils, 161 ^13^C-labeled PSMs, 36 peptides, and 26 proteins/protein groups were identified from the plants (Table 2). The median enrichment levels of ^13^C were 11% for the labeled microbial proteins and 54% for the labeled plant proteins.

The sequences of the labeled microbial proteins identified by Sipros 4 were used to infer their taxonomic origins and biological functions (Figs. 3 and 4). In the ^13^C-methanol SIP experiment, 136 labeled proteins/protein groups had phylum-level taxonomic assignments, including 77 *Proteobacteria* proteins/protein groups (391 total PSMs at 35% median ^13^C enrichment), 47 *Actinobacteriota* proteins/protein groups (105 total PSMs at 6% median ^13^C enrichment), and 5 *Acidobacteriota* proteins/protein groups (9 total PSMs at 6% median ^13^C enrichment) (Figure S2). At the order level, 58 proteins/protein groups were identified from *Rhizobiales* (333 PSMs at 32% median ^13^C enrichment), 10 proteins/protein groups from *Burkholderiales* (40 PSMs at 67% median ^13^C enrichment), and 8 proteins/protein groups from *Mycobacteriales* (13 PSMs at 6% median ^13^C enrichment). In the ^13^CO_2_ SIP experiment, 76 labeled proteins/protein groups had phylum-level taxonomic assignments, including 41 *Proteobacteria* proteins/protein groups (139 total PSMs at 11% median ^13^C enrichment), 33 *Actinobacteriota* proteins/protein groups (91 total PSMs at 14% median ^13^C enrichment), and 3 *Acidobacteriota* proteins/protein groups (7 total PSMs at 59% median ^13^C enrichment) (Figure S2). On the order level, 15 proteins/protein groups were identified from *Rhizobiales* (37 PSMs at 6% median ^13^C enrichment), 11 proteins/protein groups from *Burkholderiales* (57 PSMs at 22% median ^13^C enrichment), and 10 proteins/protein groups from *Actinomycetaless* (39 PSMs at 20% median ^13^C enrichment). The different median enrichment levels and label abundances of these taxa reflected their different ecological roles in the microbial communities.

Due to the shallow metagenome sequencing, only 54 MAGs were generated from the soil metagenomes [19]. In the ^13^C-methanol SIP experiment, Sipros 4 identified ^13^C-labeled unique proteins from 1 *Rhizobiales* MAG, 1 *Sphingomonadales* MAG, and 1 *Propionibacteriales* MAG, and 3 additional MAGs from other Orders (Supplementary Table S7). In the ^13^CO_2_ SIP experiment, Sipros 4 identified ^13^C-labeled unique proteins from 2 *Rhizobiales* MAGs, 1 *Actinomycetales* MAG, 1 *Pseudomonadales* MAG, and 5 additional MAGs from 3 other Orders (Supplementary Table S7). This demonstrated that strain-level taxonomic resolution can be obtained by combining genome-resolved metagenomes with proteomic SIP.

The functional annotations of the large number of labeled proteins identified by Sipros 4 uncovered the de novo protein synthesis activities in the labeled microorganisms using the assimilated labeled substrates. ^13^C-methanol SIP labeled 22 methanol dehydrogenases (XoxF/mxaF) proteins/protein groups (258 PSMs at 59% median ^13^C enrichment) which can convert methanol to formaldehyde. For the downstream utilization of formaldehyde, a labeled formaldehyde-activating enzyme (fae) capable of oxidating formaldehyde to CO_2_ was identified by 2 PSMs at 82% median ^13^C enrichment and two transaldolases (tal) in the ribulose monophosphate (RuMP) pathway for formaldehyde assimilation were identified by 6 PSMs at 32% median ^13^C enrichment. Furthermore, multiple enzymes in the glycolysis (EMP) pathway were labeled, including two glyceraldehyde-3-phosphate dehydrogenases (gapA) (3 PSMs at 8% median ^13^C enrichment), two enolases (eno) (4 PSMs at 92% median ^13^C enrichment), and one dihydrolipoyllysine-residue acetyltransferase (aceF) (3 PSMs at 96% median ^13^C enrichment). Many high-abundance enzymes in the citric acid cycle (TCA) were labeled by ^13^C-methanol SIP, including one aconitate hydratase (acnA) (1 PSM at 88% median ^13^C enrichment level), one isocitrate dehydrogenase (icd) (2 PSM at 5% median ^13^C enrichment level), and three malate dehydrogenases (mdh) (18 PSM at 59% median ^13^C enrichment level) (Figure S3, Supplementary Table S8).

The SIP results of Sipros 4 showed that ^13^CO_2_ SIP labeled 6 xoxF/mxaF proteins/protein groups (21 PSMs at 6% median ^13^C enrichment level), 4 proteins/protein groups (14 PSMs at 20% median ^13^C enrichment level) in the EMP pathway, 7 proteins/protein groups (8 PSMs at 9% median ^13^C enrichment level) in the TCA, and 3 ribosomal proteins/protein groups (19 PSMs at 20% median ^13^C enrichment level) (Figure S4, Supplementary Table S8). The resemblance in the proteome labeling patterns between ^13^CO_2_ SIP and ^13^C-methanol SIP suggested methanol as a key plant exudate [24, 25] transferring carbon from plants to their rhizosphere communities.

## Discussion

Our benchmarking results from the standard *E. coli* cultures and natural soil samples showed that Sipros 4 was able to identify more labeled PSM, peptides, and proteins with a greater atom% quantification precision and accuracy than alternative algorithms, including Calisp [20] and MetaProSIP [21]. The benchmarks also demonstrated the unique capability of Sipros to identify proteins with isotopic enrichment levels higher than 25%. Calisp and MetaProSIP failed on the standard *E. coli* samples with ≥ 25 atom% ^13^C, because they relied on the standard database searching tools that do not consider variable isotopic labeling during PSM identification. Peptides not identified by the standard database searching are not passed to the enrichment quantification step performed by Calisp and MetaProSIP. In contrast, the Sipros algorithm itself performs enrichment-resolved database searching over the full enrichment range and, therefore, can identify peptides with the 1 atom% enrichment increments between 0 atom% to 100 atom%.

These algorithms also employ different approaches to estimate the enrichment level of a PSM from its MS/MS data. Calisp and MetaProSIP both use the isotopic envelope of the precursor ion in the MS1 scan to estimate its atom%. Sipros 3 and 4 estimate the atom% of a PSM based on the isotopic envelopes of all the observed fragment ions in the MS2 scan. The higher performance of Sipros in enrichment quantification may be attributed to its isotopic fitting against multiple isotopic envelopes of the fragment ions, instead of a single isotopic envelope of the precursor ion. This allows aggregating multiple isotopic envelopes in the MS2 scans for atom% estimation.

A drawback of Sipros 3, in comparison to Calisp and MetaProSIP, was its higher computational cost stemming from the enrichment-resolved database searches at 101 enrichment levels. To address this, we systematically profiled and optimized the Sipros codebase to increase computational efficiency. In addition to the multi-node process-level parallelism and the multi-core thread-level parallelism, we harnessed the Single Instruction Multiple Data (SIMD) instructions in modern CPUs to enable fine-grained data parallelism on key operations. The resultant Sipros 4 can run > 20-fold faster than Sipros 3. The computational times for database searching on a commodity computer server were comparable among Sipros 4 (~ 0.5 h), Calips with Proteome Discoverer (~ 3 h), and MetaProSIP with Comet (~ 0.5 h) for the ^13^C-labeled *E. coli* datasets.

Proteomic SIP quantifies both the label abundances and the enrichment levels of the labeled proteins and, by extension, their source organisms. The enrichment level reflects the percentage of the labeled substrate, relative to the unlabeled background substrates, that were assimilated and used by an organism for amino acid synthesis. For example, in the ^13^CO_2_ SIP experiment, the median ^13^C enrichment levels were 11% for the labeled microbial proteins and 54% for the labeled plant proteins. Plants were labeled at higher ^13^C atom% probably because fixing ^13^CO_2_ and recycling the extant biomass are the only two carbon supplies for plant growth during the labeling [26]. The lower ^13^C atom% of microorganisms likely reflected their reliance on diverse types of carbon sources, encompassing the unlabeled soil organic matter and the partially labeled plant exudate [27].

The label abundances measure the relative abundances of labeled proteins and labeled organisms in terms of labeled PSM counts. For example, in the ^13^C-methanol SIP experiment, the aggregate label abundance of the soil community increased from 186 PSMs with 3 days of labeling to 428 PSMs with 8 days of labeling, while the median enrichment level of those PSMs only increased moderately from 43.5 atom% ^13^C in day 3 to 53.5 atom% ^13^C in day 8. The 2.3-fold rise of the community label abundance likely resulted from the production of new microbial proteins and the division of microbial cells over those additional 5 days of labeling [28]. The 10% increase in the median atom% may reflect a modest increase in the proportion of ^13^C methanol and its labeled derivatives used for the new biomass production.

The biological significance of the labeled proteins can be examined based on their function annotations and taxonomical assignments. Each labeled protein is a biomarker for the isotopic incorporation by its originating organism. The taxonomy of the source organisms can be inferred from the sequences of the labeled proteins (Supplementary Tables S6 and S7). In our ^13^C-methanol SIP study, methanol labeled a known methylotrophic genus, *Hyphomicrobium* [29]. In the ^13^CO_2_ SIP experiment, CO_2_ labeled known plant growth-promoting bacteria (PGPB) from *Bradyrhizobium* and *Micrococcaceae* [30]. These rhizosphere microorganisms may utilize organic acids, amino acids, or sugar from plant root exudates as a carbon source [31–33]. When coupled with genome-resolved metagenomics, the labeled proteins can directly identify which MAGs have incorporated the SIP isotope. The comprehensive functional profile of a high-quality MAG allows inferences into the larger suite of metabolic pathways involved in the uptake of the labeled substrate. This was demonstrated by the identification of the methylotrophic pathways and associated downstream pathways in the labeled MAGs in the ^13^C-methanol SIP experiment [19]. Ultimately, the label abundances and enrichment levels of the labeled proteins uncover the protein synthesis activities accompanying the metabolism of labeled substrates by different taxa, which can then be analyzed further at the community level.

The functional annotations of the labeled proteins can reveal the de novo protein synthesis activities of the source organisms, providing information to detect direct translational responses to a perturbation. As an organism assimilates the SIP isotope, it produces partially labeled amino acids for protein synthesis. Because protein synthesis accounts for 70% to 80% of the ATP budget of microorganisms [34, 35], the labeled proteome of an organism reveals which biological processes it invests its scarce energy budget into. In a competitive community, an organism should make its energy investment decisions prudently based on the anticipated future return from the present investment in light of the perceived opportunities arising from its external environment. For instance, the ^13^C-methanol SIP results showed that many methanol dehydrogenases (e.g., XoxF and MxaF) and other enzymes (e.g., tal and fae) involved in the methanol utilization were labeled during their de novo synthesis after methanol amendments (Fig. 3 and Supplementary Figure S3) [36]. Lanthanide-dependent methanol dehydrogenases (XoxF type) from *Rhizobiales* or other *Proteobacterial* taxa have been reported to contribute to the degradation of methanol in soil [37, 38]. This demonstrated that the increased methanol availability prompted the organisms to invest in the biological processes to collect and consume methanol.

The newly synthesized abundances of enzymes measured by proteomic SIP are different from the standing abundance of these enzymes measured by regular proteomics. The standing abundance of an enzyme at the end of SIP is determined by its extant unlabeled copy numbers at the beginning of SIP, plus the de novo synthesis of newly labeled copies, and minus the degradation of extant copies, over the period of SIP. The labeled proteome of the ^13^C-methanol SIP and ^13^CO_2_ SIP also included many housekeeping proteins (Fig. 4 and Figure S4), which can be attributed to the general growth of the source organisms.

## Conclusions

Our benchmarking tests demonstrated the high performance and computational efficiency of Sipros 4 for sensitive detection of labeled proteins and accurate quantification of their enrichment levels in SIP experiments. The label abundances and enrichment levels of the labeled proteins provided rich taxonomical and functional information about their source organisms. Analyses of real-world SIP experiments showcased the use of the labeled proteins to identify the microbial consumers of the labeled substrates, reconstruct their genomes, and define their de novo protein synthesis activities during the SIP labeling. Continued development of analytical tools, such as Sipros 4, greatly expanded our capacity to understand metabolic activities in complex microbial communities by directly linking substrate assimilation with phylogeny and functions.

## Materials and methods

### Preparation of the *E. coli* standard samples with known ^13^C atom%

Each *E. coli* culture with a pre-defined ^13^C incorporation level was grown in a defined medium in the following steps. First, 5 μL of *E. coli* DH5α (New England Biolabs) was inoculated into 10 mL of LB medium and incubated for 1 day at 37 °C in an incubator (Robbins Scientific). Next, 700 μL *E. coli*-LB culture solution was mixed with 300 μL 50% glycerin in a 1-mL centrifuge tube and stored at − 80 °C as a new inoculant. Then, the 1-mL *E. coli* inoculant was washed twice with 1 mL of PBS buffer, and 5 μL of the washed cells in PBS buffer was inoculated into 10 mL of a ^13^C-labeled M9 growth medium in a 50-mL centrifuge tube in a biological safety cabinet (Thermo Scientific). Supplementary Table S9 lists the recipes of the M9 growth media at different ^13^C atom%, including the amounts of ^12^C glucose and ^13^C glucose (D-Glucose-^13^C_6_, ≥ 99 ^13^C atom%, Sigma-Aldrich) as the sole carbon source for bacterial growth. Finally, the ^13^C-labeled culture was incubated at 37 °C for 2 days, reaching > 0.5 OD 600. Three replicate cultures were grown for each ^13^C atom%.

### Protein extraction and LC–MS/MS

The protein extraction was performed as described previously [19] with some minor modifications. A cell pellet in 5 mL of a ^13^C-labeled *E. coli* culture was harvested immediately after centrifugation at 4 °C and 10,000 × *g* for 1 min. The pellet was washed twice with 1 mL of 10 mM Tris–HCl buffer at pH 7.0 and resuspended in 0.5 mL of lysis buffer (100 mM Tris–HCl, 4% SDS, and 0.1 M freshly added DTT). The cells were sonicated on ice for five cycles of 30 s each with pulses. After centrifugation at 4 °C and 10,000 × *g* for 1 min, the supernatant was collected and mixed with 0.5 mL of chilled (− 20 °C) 50% TCA to reach a final concentration of 25% TCA. The tubes were stored at − 20 °C overnight to precipitate the proteins. The samples were centrifuged at 20,800 × *g* for 20 min to collect the protein pellets, which were then washed twice with 1 mL of chilled (− 20 °C) 80% acetone and once with 1 mL of chilled acetone. After each wash step, the samples were centrifuged again at 20,800 × *g* for 20 min to pellet down the proteins. The protein pellets were dried in a centrifugal evaporator and resolubilized in 585 μL of urea-Tris–HCl solution (8 M urea, 0.1 M Tris–HCl, pH 8.0). An aliquot of 3 μL of fresh 1 M DTT solution was added to the urea-Tris–HCl solution to reach a final concentration of ~ 5 mM DTT. The mixture was vortexed for 20 min to dissolve the protein. Bubbles were removed by centrifugation at 10,000 × *g* for 10 min. Then, an aliquot of 12 μL of 1 M iodoacetamide solution was added to the mixture to achieve an iodoacetamide concentration of 20 mM. After vortexing for 10 s, the solution was incubated in the dark at room temperature for 30 min. After centrifugation at 10,000 × *g* for 5 min, the supernatant was divided into three 1 mL tubes, allocating 200 μL for each. The protein concentration was measured using the Pierce BCA Protein Assay Kit (Thermo Scientific).

Protein digestion was performed using the FASP method [39] in a 1-mL 30-kDa ultrafiltration unit (Vivacon 500, Sartorius). Each sample aliquot with 50 μg of protein was digested overnight at 37 °C with 2 μg of sequencing-grade modified trypsin (V5113, Promega). The peptide digest was desalted by Pierce Peptide Desalting Spin Columns (Thermo Scientific) and its concentration was measured by NanoDrop 2000 Spectrophotometers (Thermo Scientific). The peptide separation was performed by reverse-phase XSelect CSH C18 2.5 μm resin (Waters) on a 150 × 0.075 mm column using an UltiMate 3000 RSLCnano system (Thermo Scientific) with 1 μg of peptides. The peptides were eluted with a 90-min gradient from 98% solution A and 2% solution B to 65% solution A and 35% solution B (solution A = 0.1% formic acid, 0.5% acetonitrile and 99.4% water; and solution *B* = 0.1% formic acid and 99.9% acetonitrile). The eluted peptides were ionized by electrospray (2.4 kV) and analyzed by an Orbitrap Fusion Tribrid mass spectrometer (Thermo Scientific) in the data-dependent acquisition mode. MS1 data were acquired using the Orbitrap analyzer in the profile mode at a resolution of 120,000 over the m/z range of 375–1500. MS2 data were acquired using the Orbitrap analyzer in the centroid mode at a resolution of 30,000 after HCD activation. The precursor isolation window for MS2 was set to 5 in width. Dynamic exclusion time was set to 20 s, exclude isotope was set to true, and mass tolerance of the isolation window was set to 10 ppm. The HCD energy was set to 28% for precursors with charge states between + 3 and + 7 and precursors in the m/z range of 375–650. The HCD energy was set to 31% for precursors with a charge state of + 2 and precursors in the m/z range of 650–1500. Precursors with an unknown charge state or a charge state lower than + 2 or higher than + 7 were excluded from the MS2 selection.

### MS/MS data extraction

The mass spectrometry data need to be extracted into the FT1/FT2, MS1/MS2, or mzML formats as the input for Sipros. The RAW files generated from the ^13^C-labeled *E. coli* analyses were converted into FT1 and FT2 files using Raxport (https://github.com/thepanlab/Raxport.net) on a Linux server running CentOS 7. Raxport was upgraded to be compatible with both Linux and Windows by using the RawFileReader library (Thermo Scientific) with the Mono framework (https://www.mono-project.com/). The RAW files for the standard *E. coli* samples were uploaded to the ProteomeXchange repository under the access number PXD041414.

The RAW files for the ^15^NH_4_Cl-labeled acid mine drainage (AMD) community were generated in a previous study [22] and were uploaded to the ProteomeXchange repository with the accession number PXD041958. The RAW files for the ^13^C-methanol-labeled soil communities and the ^13^CO_2_-labeled soil communities [19] were downloaded from the ProteomeXchange repository under the access numbers of PXD011738 (unlabeled initial soil), PXD011739 (^13^C-methanol-labeled soil), PXD011737 (^13^CO_2_-labeled Arabidopsis rhizosphere soil), PXD011891 (^13^CO_2_-labeled maize rhizosphere soil), and PXD011892 (^13^CO_2_-labeled wheat rhizosphere soil). All these RAW files were converted to FT1 and FT2 files using Raxport.

### Algorithmic improvements in Sipros 4 for SIP searches

By default, Sipros 4 performs database searching across 101 atom% levels, ranging from 0 to 100% in 1% increments, as specified in the configuration files. Subsequently, the PSMs at these pre-defined integer atom% levels were filtered based on their scores to reach a certain FDR level. Users may customize the atom% increment (e.g., 0.5%) and the search range (e.g., from 0 to 10%) in the configuration file based on their experimental requirements. A PSM identifies a peptide at an atom% level that best explains the corresponding MS/MS spectrum. The most abundant isotopic mass of a peptide candidate is approximated by the sum of the most abundant isotopic masses of all its residues. A series of precursor mass tolerance windows are opened for a range of unit mass offsets from the measured MS/MS precursor mass. In Sipros 4, the unit mass offset range is customized according to the enrichment level of the SIP searches based on the simulation results using the poly-Averagine peptides [40]. The size of the mass tolerance windows is configurable by users with a default value of ±0.01 Da for Orbitrap mass spectrometers. Sipros selects the peptide candidates for an MS/MS spectrum using its precursor mass tolerance windows.

To reconstruct the theoretical spectrum of a peptide candidate at a given atom%, Sipros computes the isotopic envelopes of all B and Y ions from this peptide. To speed up this computation task, the polynomial expansion algorithm used in Sipros 3 was replaced with the convolution algorithm [41] in Sipros 4. The convolution algorithm was vectorized using single instruction multiple data (SIMD) provided by the omp simd directive in OpenMP 4.0. The SIMD parallelism in Sipros 4 accelerated the convolution computation on individual CPU cores on top of the thread-level parallelism on multi-core CPUs and the process-level parallelism across computer nodes implemented in Sipros 3.

The scoring function was optimized in Sipros 4 to improve the performance of PSM identification. The score for a PSM, $p$, is a sum of the scores of the $n$ B/Y ions found in an observed MS/MS spectrum:

1 $$p=\sum_{k=1}^{n}{{s}_{k}c}_{k}{h}_{k}{g}_{k}$$

where, for the *k*^th^ matched B/Y ion, ${h}_{k}$ is the mass accuracy score defined in Eq. 2, ${s}_{k}$ is the isotopic envelope score defined in Eq. 3, ${c}_{k}$ is the charge state penalty, and ${g}_{k}$ is the complementary fragment penalty. ${c}_{k}$ takes a value of 1 when the expected charge state matches the observed charge state; otherwise, it assumes a value of 0.5. ${g}_{k}$ is assigned a value of 2 in the presence of the complementary fragment ion; otherwise, it is set to 1.

The mass accuracy score of the *k*^th^ matched B/Y ion, ${h}_{k}$, is defined as:

2 $${h}_{k}=2\left[1-pnorm\left(0, t/2, {m}_{k}\right)\right]$$

where $pnorm\left(\bullet \right)$ is the cumulative density function (CDF) at the threshold of ${m}_{k}$ of a normal distribution with the mean of 0 and the standard deviation of $t/2$, $t$ is the fragment mass tolerance defined by the user in the configuration file, and ${m}_{k}$ represents the observed average mass error of the isotopic peaks of the matched B/Y ion.

The isotopic envelope score of the *k*^th^ matched B/Y ion, ${s}_{k}$, is computed as

3 $${s}_{k}=1+{\sum }_{i}{e}_{i}-{\sum }_{j}{u}_{j}$$

where ${e}_{i}$ is the reward for finding an expected isotopic peak $i$ (Eq. 4) in this fragment ion's isotopic distribution and ${u}_{j}$ is the penalty for missing an expected isotopic peak $j$ in this fragment ion's isotopic distribution (Eq. 5).

4 $${e}_{i}=g\bullet \left[1-erf\left(\frac{\left|{x}_{i}-{y}_{i}\right|}{\sqrt{{x}_{i}^{2}+{y}_{i}^{2}}}\right)\right]$$

5 $${u}_{j}={x}_{j}\bullet \left[a+b\times {\left(q-50\%\right)}^{c}\right]$$

where $x$ is the expected relative intensity, $y$ is the observed relative intensity matched within the mass error tolerance, $erf\left(\bullet \right)$ is the Gauss error function [42], and $q$ represents the isotopic atom% being searched. The constants, $a$, $b$, $c$, and $g$, were set to 0.005, 4, 8, and 0.5, respectively, based on heuristic optimization and are user-configurable in the search configuration file.

The code and user manual of Sipros 4 were released at https://github.com/thepanlab/Sipros4.

### Database searching of SIP samples by Sipros 4

The FT2 files of the *E. coli* samples at different ^13^C atom% levels were searched against a target-decoy protein sequence database comprised of the *Escherichia coli* (strain K12) proteome from UniProt and the non-*E. coli* contaminant proteins from https://www.thegpm.org/crap/. The reverse sequences of these target proteins were added to the database as decoys. The mass error tolerance was set to 0.01 Da for precursors and fragments. The false discovery rate (FDR) of peptide identifications was controlled to 1% by adjusting the score thresholds of PSMs. Protein identifications were filtered to reach 1% FDR based on the highest PSM score for protein identification. At least one unique peptide was required for each identified protein/protein group.

The ^15^NH_4_Cl-labeled AMD datasets were processed similarly using Sipros 4. The target-decoy protein sequence database was constructed from the AMD metagenome assemblies [22]. The SIP isotope was changed to ^15^N. The mass error tolerance was set to 0.05 for precursors and 0.02 for fragments. The FDRs of peptides and proteins were all controlled to 1% as described above.

For the analysis of the low-resolution ion trap MS2 data from the ^15^N-labeled spiked mouse gut microbiome sample [23], the mass error tolerance was set to 0.02 for precursors and 0.11 for fragments. In this low-resolution MS2 setting, Sipros used the most intense peak within each isotopic envelope to score PSMs, as opposed to all isotopic peaks in the high-resolution MS2 setting, which reduced the performance of Sipros.

Regular label-free searches were performed on the soil ^13^C-methanol and ^13^CO_2_ SIP datasets using Sipros Ensemble [43] against a protein database containing all predicted proteins from the soil metagenome assemblies. All the identified proteins were used to construct the protein database for ^13^C SIP searches using Sipros 4. Protein sequences of Arabidopsis, wheat, and maize were also added to the protein database for the ^13^CO_2_ SIP datasets. The mass error tolerance was set to 0.05 for precursors and 0.02 for fragments. The MS/MS spectra containing high-density clusters of noise peaks (i.e., > 255 peaks within any 250-wide m/z window of a spectrum) were removed.

The database search for the *E. coli* samples and AMD samples was performed on a compute node equipped with dual 22-core Intel Xeon CPUs (Gold 6152) and 376 GB system memory on the Schooner supercomputer and on a computer server equipped with 24-cores AMD Ryzen CPU (5965WX) and 512 GB system memory. The database search for the soil samples was completed on computing nodes equipped with dual 10-core Intel Xeon Haswell CPUs and 32 GB system memory on the Schooner supercomputer.

### SIP analysis by Calisp and MetaProSIP

Calisp and MetaProSIP can quantify the atom% of peptides that have been identified by label-free database searching using Proteome Discoverer (for Calisp) or Comet (for MetaProSIP). The RAW files of all the SIP samples were converted into the mzml format using ProteoWizard. The regular database searching was performed with Proteome Discoverer using the default parameters from its data-dependent acquisition workflow template. The same protein databases described above were provided to Proteome Discoverer. The FDRs of identified PSMs, peptides, and proteins were all controlled to 1%. The enrichment levels of the identified peptides were quantified by Calisp using the default parameters according to its tutorial (https://sourceforge.net/p/calis-p/wiki/Home/).

The SIP analysis by MetaProSIP was conducted in the TOPASS environment by OpenMS [44]. Briefly, the MS/MS data of all the SIP samples were converted to mzml files and searched using Comet [45] with default parameters to generate label-free identifications. The FDRs of identified PSMs, peptides, and proteins were controlled to 1%. The label-free identifications and mzml files were provided to MetaProSIP as the input using default parameters according to https://sourceforge.net/projects/open-ms/files/Papers/MetaProSIP/.

### Analysis and visualization of the SIP search results

The eggNOG-mapper [46] was used to annotate the functions of the proteins based on GO terms, EC numbers, and KEGG terms. The taxonomy of protein was annotated using the annoTree database [47] with DIAMOND [48] and MEGAN6 [49]. A protein group was assigned to a taxon if more than 70% of its member proteins were assigned to this taxon. Similarly, a protein group was annotated with a functional assignment if more than 70% of its member proteins were annotated with this functional assignment. For the genome-resolved proteomic SIP analysis, an MAG was marked as labeled if at least one unique labeled peptide was identified from this MAG. Functional enrichment analysis of labeled proteins was performed using clusterProfiler [50]. The phylogenetic tree of labeled microorganisms was visualized using ggtree [51]. The Student’s t-test and Wilcoxon test were performed in R 4.2.1 [52].

### Supplementary Information

Additional file 1: Supplementary Table S1. Comparison of different isolation windows sizes for 50% 13C-labeled E. coli.

Additional file 2: Supplementary Table S2. Comparison of Sipros 4 and Sipros 3 using standard 13C-labeled E. coli samples.

Additional file 3: Supplementary Table S3. Unlabeled regular search results of Sipros Ensemble, Proteome Discoverer 3.0, and MaxQuant 2.0 of standard 13C-labeled E. coli samples.

Additional file 4: Supplementary Table S4. Comparison of Sipros 4 and Sipros 3 using the standard 15N-labeled acid mine drainage samples.

Additional file 5: Supplementary Table S5. Nonredundant reference peptides and ^15^N-labeled peptides from mouse stool samples.

Additional file 6: Supplementary Table S6. Unlabeled PSMs, peptides, and proteins identified in the initial soil,13C-methanol SIP soil and 13CO2 SIP soil.

Additional file 7: Supplementary Table S7. Genome-resolved proteomic SIP results.

Additional file 8: Supplementary Table S8. Labeled protein identifications from ^13^C-methanol and ^13^CO2 SIP.

Additional file 9: Supplementary Table S9. Recipes for ^13^C-labeled M9 media for standard E. coli.

Additional file 10: Supplementary Figure S1.Venn diagrams of the proteins identified by Sipros 3, Sipros 4, Calisp, and MetaproSIP on E. coli standard samples. Supplementary Figure S2. Taxonomic tree of the microbial proteins identified in the initial soils, 13Cmethanol SIP soils, and 13CO2 SIP soils. The tree tips represent the inferred Orders of identified proteins. The tree branches are colored based on the Phylum-level classification. The four bar charts from the left to the right represent the number of unlabeled proteins identified in the 13C-methanol SIP soils, the number of unlabeled proteins identified in the 13CO2 SIP soils, the number of labeled proteins identified in the 13C-methanol SIP soils, and the number of labeled proteins identified in the 13CO2 SIP soils from each Order. The heatmap columns from the left to right show the average enrichment levels of the labeled proteins identified in the 13C-methanol SIP soils and the 13CO2 SIP soils from each Order. Supplementary Figure S3. functional analysis of 13C-methanol SIP results. (A) Boxplot of the 13C enrichment levels of PSMs identified in the day-3 sample and the day-8 sample. The t-test *p*-value is less than 0.001, indicated by ***. (B) Boxplot of the labeled protein counts identified in the day-3 sample and the day-8 sample. The t-test *p*-value is less than 0.05, indicated by *. (C) 13C-labeled enzymes involved in methanol degradation. The names of the pathways are highlighted in blue. The enzyme names and EC numbers are annotated in yellow for identified enzymes and in red for identified enzymes significantly enriched in the 13C-labeled proteins. (D) Top-10 enriched KEGG Orthology (KO) terms with adjusted *P*-value < 0.01 for the 13C-labeled proteins. (E) Enriched molecular functions of GO terms, with adjusted *P*-value < 0.01, for the 13Clabeled proteins. Supplementary Figure S4. functional analysis of 13CO2 SIP results. (A) total label abundances of the plant proteins and microbial proteins. The t test *p*-value is less than 0.001, indicated by ***. (B) Top-10 enriched KO terms with adjusted *P*-value < 0.01 for the 13C-labeled proteins. (C) Enriched molecular functions of GO terms, with adjusted *P*-value < 0.01, for the 13C-labeled proteins.

## Data Availability

No datasets were generated or analysed during the current study.
