# Supplementary material for: Proteomic stable isotope probing with an upgraded Sipros algorithm for improved identification and quantification of isotopically labeled proteins
Source: Microbiome. 2024 Aug 8;12:148. doi: 10.1186/s40168-024-01866-1 (PMC11313024; doi:10.1186/s40168-024-01866-1)
Supplement: Supplementary file 11 — Additional file 10: Supplementary Figure S1.Venn diagrams of the proteins identified by Sipros 3, Sipros 4, Calisp, and MetaproSIP on E. coli standard samples. Supplementary Figure S2. Taxonomic tree of the microbial proteins identified in the initial soils, 13Cmethanol SIP soils, and 13CO2 SIP soils. The tree tips represent the inferred Orders of identified proteins. The tree branches are colored based on the Phylum-level classification. The four bar charts from the left to the right represent the number of unlabeled proteins identified in the 13C-methanol SIP soils, the number of unlabeled proteins identified in the 13CO2 SIP soils, the number of labeled proteins identified in the 13C-methanol SIP soils, and the number of labeled proteins identified in the 13CO2 SIP soils from each Order. The heatmap columns from the left to right show the average enrichment levels of the labeled proteins identified in the 13C-methanol SIP soils and the 13CO2 SIP soils from each Order. Supplementary Figure S3. functional analysis of 13C-methanol SIP results. (A) Boxplot of the 13C enrichment levels of PSMs identified in the day-3 sample and the day-8 sample. The t-test p-value is less than 0.001, indicated by ***. (B) Boxplot of the labeled protein counts identified in the day-3 sample and the day-8 sample. The t-test p-value is less than 0.05, indicated by *. (C) 13C-labeled enzymes involved in methanol degradation. The names of the pathways are highlighted in blue. The enzyme names and EC numbers are annotated in yellow for identified enzymes and in red for identified enzymes significantly enriched in the 13C-labeled proteins. (D) Top-10 enriched KEGG Orthology (KO) terms with adjusted P-value < 0.01 for the 13C-labeled proteins. (E) Enriched molecular functions of GO terms, with adjusted P-value < 0.01, for the 13Clabeled proteins. Supplementary Figure S4. functional analysis of 13CO2 SIP results. (A) total label abundances of the plant proteins and microbial proteins. The t te [file 40168_2024_1866_MOESM10_ESM.pdf]

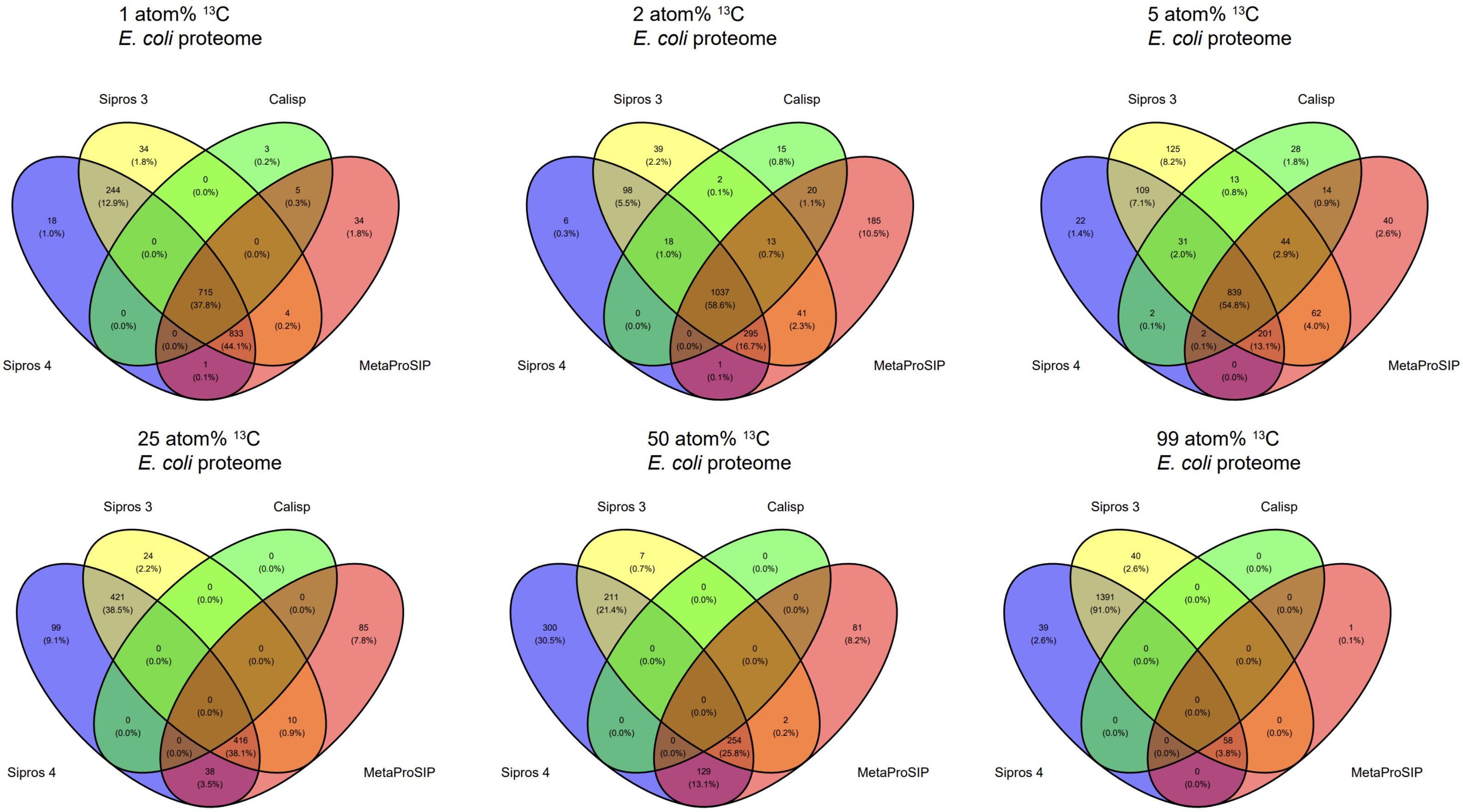

Supplementary figure S1: Venn diagrams of the proteins identified by Sipros 3, Sipros 4, Calisp, and MetaproSIP on *E. coli* standard samples.

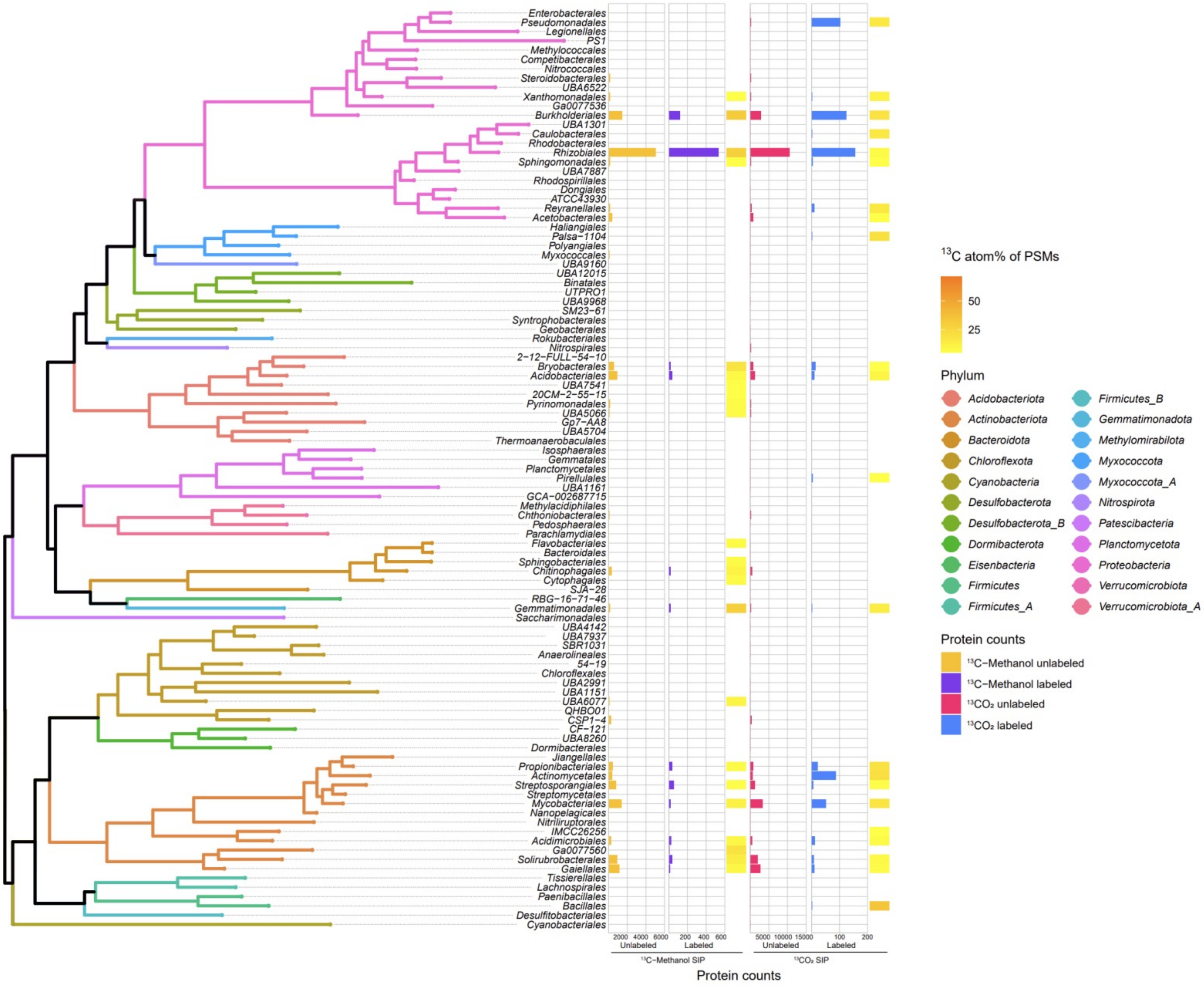

Supplementary figure S2: Taxonomic tree of the microbial proteins identified in the initial soils, ^{13}C-methanol SIP soils, and $^{13}CO_2$ SIP soils. The tree tips represent the inferred Orders of identified proteins. The tree branches are colored based on the Phylum-level classification. The four bar charts from the left to the right represent the number of unlabeled proteins identified in the ^{13}C-methanol SIP soils, the number of unlabeled proteins identified in the $^{13}CO_2$ SIP soils, the number of labeled proteins identified in the ^{13}C-methanol SIP soils, and the number of labeled proteins identified in the $^{13}CO_2$ SIP soils from each Order. The heatmap columns from the left to right show the average enrichment levels of the labeled proteins identified in the ^{13}C-methanol SIP soils and the $^{13}CO_2$ SIP soils from each Order.

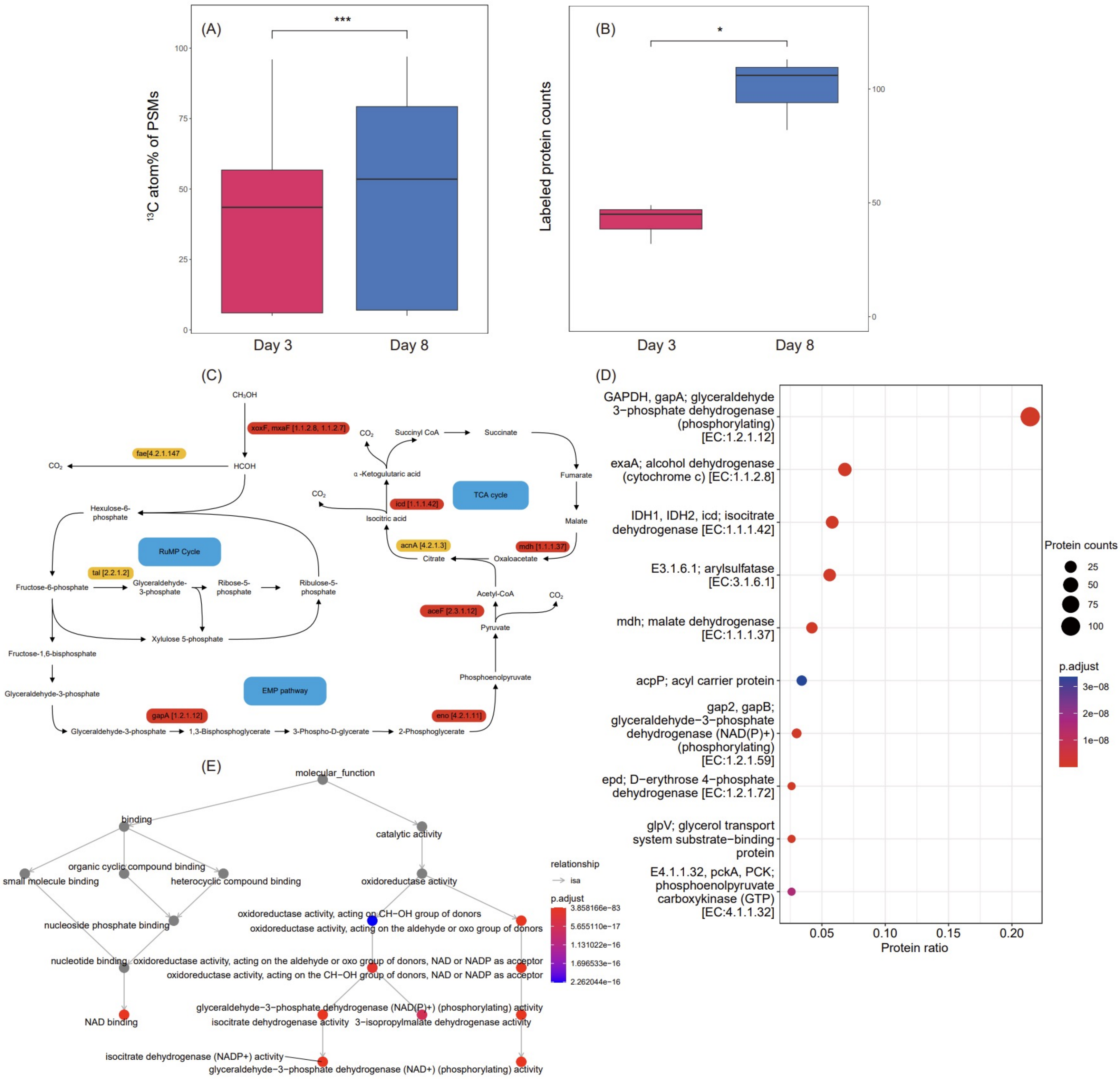

Supplementary figure S3: functional analysis of ^{13}C-methanol SIP results. (A) Boxplot of the ^{13}C enrichment levels of PSMs identified in the day-3 sample and the day-8 sample. The t-test p-value is less than 0.001, indicated by ***. (B) Boxplot of the labeled protein counts identified in the day-3 sample and the day-8 sample. The t-test p-value is less than 0.05, indicated by *. (C) ^{13}C-labeled enzymes involved in methanol degradation. The names of the pathways are highlighted in blue. The enzyme names and EC numbers are annotated in yellow for identified enzymes and in red for identified enzymes significantly enriched in the ^{13}C-labeled proteins. (D) Top-10 enriched KEGG Orthology (KO) terms with adjusted P-value < 0.01 for the ^{13}C-labeled proteins. (E) Enriched molecular functions of GO terms, with adjusted P-value < 0.01, for the ^{13}C-labeled proteins.

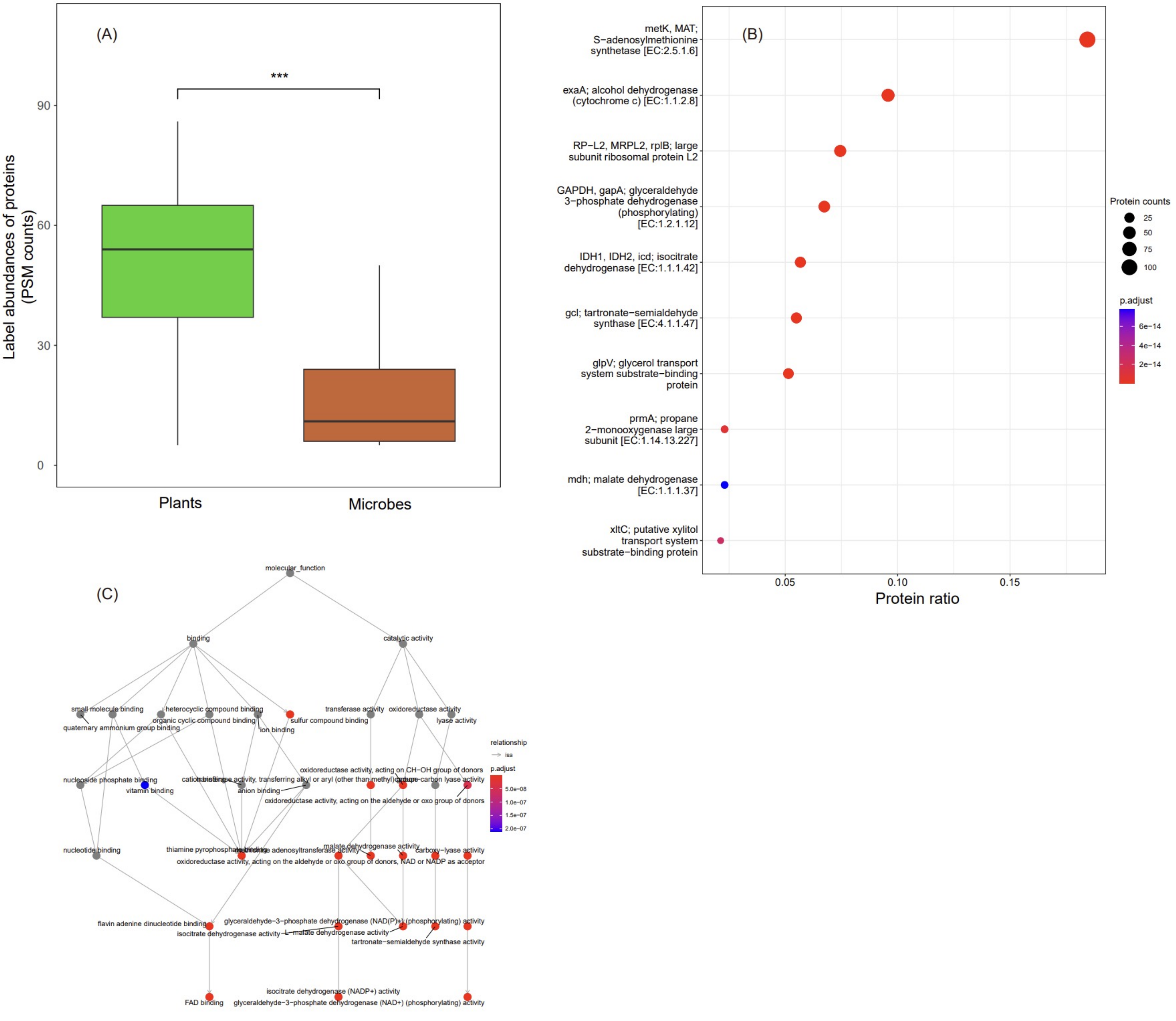

Supplementary figure S4: functional analysis of $^{13}CO_2$ SIP results. (A) total label abundances of the plant proteins and microbial proteins. The t test p-value is less than 0.001, indicated by ***. (B) Top-10 enriched KO terms with adjusted P-value < 0.01 for the ^{13}C-labeled proteins. (C) Enriched molecular functions of GO terms, with adjusted P-value < 0.01, for the ^{13}C-labeled proteins.
